# Effectiveness of multi-professional educational interventions to train Comprehensive Geriatric Assessment (CGA) – a Systematic Review

**DOI:** 10.5334/ijic.7549

**Published:** 2023-08-14

**Authors:** Sonja Lindner-Rabl, Katrin Singler, M. Cristina Polidori, Carolin Herzog, Eleftheria Antoniadou, Gerald Seinost, Regina Roller-Wirnsberger

**Affiliations:** 1 Medical University of Graz, Department of Internal Medicine, Graz, Austria; 2 Institute for Biomedicine of Ageing, Friedrich-Alexander University Erlangen-Nürnberg, Nuremberg, Germany; 3 Department of Geriatric Medicine, Klinikum Nürnberg, Paracelsus Private Medical University, Nürnberg, Nuremberg, Germany; 4 Faculty of Medicine and University Hospital Cologne, Ageing Clinical Research, Department II of Internal Medicine and Center for Molecular Medicine Cologne, Cologne, Germany; 5 Cologne Excellence Cluster on Cellular Stress-Responses in Aging-Associated Diseases (CECAD), University of Cologne, Faculty of Medicine and University Hospital Cologne, Cologne, Germany; 6 Rééducation Gériatrique, Centre Hospitalier du Nord, Luxembourg; 7 Medical University of Graz, Department of Internal Medicine, Division of Angiology, Graz, Austria

**Keywords:** integrated care, Comprehensive Geriatric Assessment, education & training, interprofessional education, ageing

## Abstract

**Introduction:**

As the world population ages, health and social care professionals are increasingly confronted with patients with chronic long-term conditions and multimorbidity, requiring an extensive assessment and integrated care management strategy. The aim of this paper was to systematically collect and assess evidence of interprofessional education and training strategies for Comprehensive Geriatric Assessment (CGA) to build a competent health workforce.

**Methods:**

A systematic review was conducted according to PRISMA guidelines and the databases Medline, CINAHL, Cochrane and Embase were searched for studies illustrating effectiveness of educational interventions for teaching and training CGA in an interprofessional context.

**Results:**

Based on 21 identified studies, a great variability and heterogeneity in duration, setting and design of the interventions was identified. Promising results were found in the domains analysed, ranging from knowledge and skills; practices and behaviour; patient health outcomes; attitudes and perceptions to collaboration and quality of care.

**Discussion:**

Education and training of transversal skills within a continuous learning approach is key to equip the health care workforce for successful CGA performance in an interprofessional environment.

**Conclusion:**

Further research in this field is recommended to strengthen the evidence-base towards development of a resilient and integrated health care workforce for an ageing population.

## Abstract (de)

**Hintergrund:**

Aufgrund der zunehmenden Alterung der Weltbevölkerung sehen sich Fachkräfte des Gesundheits- und Sozialwesens immer häufiger mit Patient*innen mit chronischen Erkrankungen (bzw. Langzeiterkrankungen) und Multimorbidität, welche eine umfassende Beurteilung und eine integrierte Versorgungsmanagementstrategie erfordern, konfrontiert. Ziel der vorliegenden Arbeit war es, systematisch Evidenz für interprofessionelle Aus- und Weiterbildungsstrategien für ein Comprehensive Geriatric Assessment (CGA) zu sammeln und zu bewerten, um Kompetenz im Gesundheits- und Sozialwesen zu generieren.

**Methodik:**

Es wurde eine systematische Übersichtsarbeit gemäß den PRISMA-Richtlinien durchgeführt und die Datenbanken Medline, CINAHL, Cochrane und Embase im Zuge dessen nach Studien durchsucht, welche die Wirksamkeit von Bildungsmaßnahmen zur Vermittlung und Ausbildung von CGA in einem interprofessionellen Kontext belegen.

**Ergebnisse:**

Basierend auf 21 identifizierten Studien wurde eine große Variabilität und Heterogenität in Bezug auf Dauer, Setting und Design der Interventionen festgestellt. Vielversprechende Ergebnisse wurden in den analysierten Bereichen gefunden, welche von Wissen und Fähigkeiten, Praktiken und Verhalten, gesundheitlichen Ergebnissen für Patient*innen, Einstellungen und Wahrnehmungen bis hin zu Zusammenarbeit und Qualität der Versorgung reichen.

**Diskussion:**

Die Aus- und Weiterbildung von transversalen Fähigkeiten im Rahmen eines kontinuierlichen Lernansatzes ist der Schlüssel dazu, Angehörige der Gesundheits- und Sozialprofessionen mit entsprechendem Werkzeug auszustatten, um geriatrische Assessments (CGA) erfolgreich im interprofessionellen Umfeld durchführen zu können.

**Schlussfolgerung:**

Es besteht der Bedarf für weitere Forschungsvorhaben in diesem Bereich, um die Evidenzbasis für die Entwicklung eines zuverlässigen, integrierten Gesundheitspersonals für eine alternde Bevölkerung zu stärken.

## (1) Introduction

Currently, European citizens reach an average life expectancy of 81 years at birth [1]. However, living longer does not necessarily entail a good quality of life in older age [2]. Actually, older adults represent a diverse population, characterized by varying cognitive and physical capacity as well as divers multi-morbidity patterns [3]. Additionally, the ageing process itself results in rising individual vulnerability related to complex and chronic health and social care needs of this population [3,4]. There is, in fact, an evidence-based demand for person-centered and tailored medical and social care provision based upon individual capacity and, therefore, strengthening of people’s and patients’ resilience [5].

In this regard, the Comprehensive Geriatric Assessment (CGA) constitutes a multi-dimensional diagnostic tool to evaluate the clinical, functional and psychosocial status of geriatric patients and subsequently coordinate and monitor clinical and social care needs and interventions targeting individual resilience [6]. Studies have demonstrated the effectiveness of CGA towards individual patients’ quality of life and health outcomes as well as towards a reduction of caregiver burden [7,8,9,10].

Due to the multidimensional nature of the CGA, an inter-professional and team-based approach is inherent for its effective and efficient provision in daily practice. The concept of integrated care bundles coherent methods and models to produce collaboration, connectivity and coordination across professional and organizational boundaries [11]. Even if integrated care is characterized by its complexity and the different forms and models it encompasses, it constitutes an asset for geriatric care in general and CGA in particular, laying the focus on multi-professional teams or care networks in terms of horizontal integration [12], in order to collaboratively meet older patients’ needs.

The World Health Organization (WHO) advocates for policy strategies strengthening health and social workforce development, with a special focus on collaborative practice [13,14]. In this context inter-professional education (IPE), meaning that

“students from two or more professions learn about, from and with each other to enable effective collaboration and improved health outcomes” [15],

has proven to facilitate team-based approaches, preparing students for person-centered care delivery for an ageing population. Despite a growing body of evidence of research in this area, conceptualization, implementation and evaluation of inter-professional educational interventions remain topics in need for further investigation [16]. Against this background, there have been different educational approaches to train staff for this collaborative care approach using CGA in daily practice. There seems more recent expert consensus, that training inter-professional health and social care teams to deliver CGA effectively ideally takes place within different inter-professional pedagogical models, such as standardized patients or client situations in practice settings amongst others. Confronting students of different professional background with the authentic complexity of older patients’ care needs has been proven an effective approach to learning to work cooperatively [17].

Based on the current situation it was the aim of this review to pull together the most recent evidence on inter-professional education and training interventions to teach workforce from different disciplines, thereby facilitating coordinated delivery of CGA in daily practice.

## (2) Methodology

This systematic review was conducted in line with the Preferred Reporting Items for Systematic Reviews and Meta-Analyses (PRISMA) 2020 checklist [18,19]. The study had been preregistered in the international prospective register of systematic reviews (PROSPERO, ID CRD42022347656) and a research protocol is accessible as supplementary material. The databases Medline (via PubMed), CINAHL (via EBSCOhost), Cochrane (via Ovid) and Embase (via Ovid) were searched for educational intervention studies in English or German language. The detailed search strategy for each database may be retrieved from the supplementary material. If applicable, controlled vocabulary terms like Medical Subject Headings (MeSH) were applied and adjusted according to specific database options. Google scholar was searched for additional grey literature (first 100 results) and further studies were determined by manual reference tracking of included studies. One author (SLR) conducted title-/abstract screening and subsequent full-text screening was performed by two authors independently (RRW, SLR). Any disagreements were solved by consulting a third reviewer (CH).

The search was limited to publications between January 2000 and September 2022 since contemporary studies rather respond to current evidence-based didactic and pedagogic approaches, thereby also taking into account possible e-learning and digitalization methods.

Bibliographic management and deduplication was carried out with the bibliographic software Endnote and manually.

In order to be included, studies had to meet the following inclusion criteria: 1) Experimental or quasi-experimental educational intervention studies or cohort studies; 2) applying a multi-professional, undergraduate and/or continuous professional development (CPD) education and training approach in the field of CGA in any setting; 3) and carrying out a self-assessed or objective evaluation of the intervention. Studies were excluded, if the intervention addressed professional silos only and focused on geriatric care in general without referring to geriatric assessments.

Outcomes of interest were attitudes and perceptions of learners; knowledge, confidence, competence, abilities or skills; learner’s behaviour and practices; as well as patients’ health outcomes.

Each included article was assessed for study quality by two independent reviewers (SLR, CH) using the Medical Education Research Study Quality Instrument (MERSQI), a tool developed particularly to assess educational studies consisting of six different domains with an overall possible score range from 0 to 18 [20]. For assessing quality of studies within this review, the tool was slightly adapted by adding the possibility to tick the answer “not applicable/could not assess” for all items if information required could not be retrieved from the article under study. Independency of raters was ensured by local separation of the reviewers. After evaluation, mean score for each article and range of all articles were calculated with MS Office Excel 2016 software and analysed. No cut-off point was determined for differentiating “high-quality” studies from “low-quality” studies.

Narrative synthesis of the data was performed, as no meta-analysis was carried out due to expected heterogeneity of outcome data.

## (3) Results

**Figure 1:**
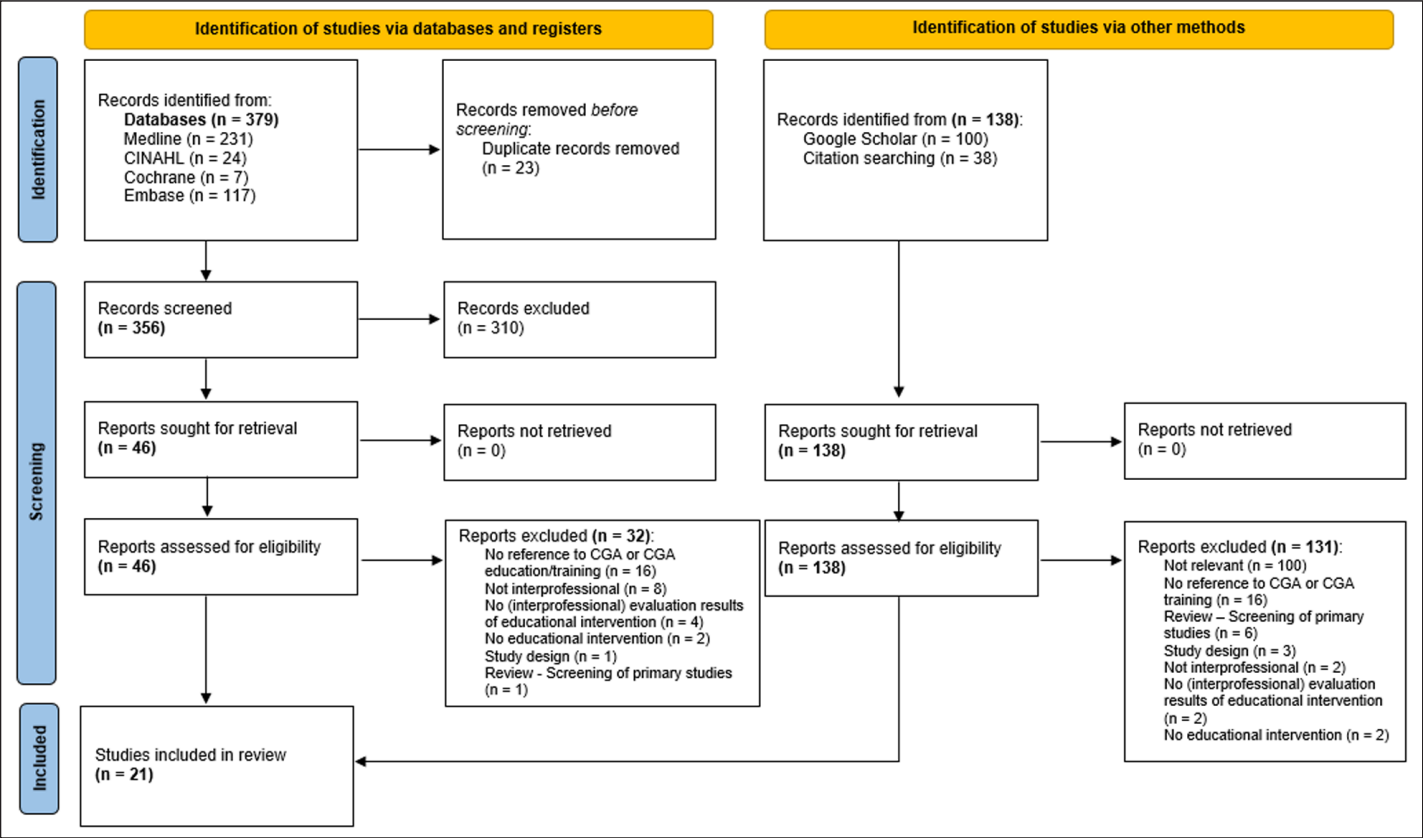
**PRISMA 2020 flow diagram.** Figure illustrates the research and screening process applied. A total of 21 studies met eligibility criteria and were included for final evaluation.

The applied search strategy yielded a total of 379 results, 138 additional results were identified on Google Scholar and via reference tracking. After deduplication (n = 23 results removed), title-/abstract screening (n = 310 results removed) and full-text screening (n = 163 results removed), 21 results were included for synthesis. The PRISMA 2020 flow diagram illustrates the screening process and demonstrates reasons for exclusion (Figure 1).

### (3.1) Study Characteristics

Table 1 (supplementary material) summarizes the 21 studies. As illustrated, these studies had been published between 2009 and 2021 with the majority being published between 2013 and 2018. Nearly all of the studies (n = 18; 85.7%) were conducted in the USA, the rest was undertaken in Greece [21], Australia [22] and Germany [23].

Included studies represented almost consistently quasi-experimental studies, either with a pre-/post-design [21,24,25,26,27,28,29,30,31,32,33], post-test only [22,34,35,36,37,38,39] or a combination of both [40,41], with the exception of one randomized controlled trial [23].

The majority of the studies (n = 17; 80.9%) considered for this review addressed undergraduate health and social care professions’ students whereas two studies evaluated educational interventions on a CPD level [21,27]. Two papers used a mixed-population approach with undergraduate students as well as health and social care professionals alike [37,41].

Disciplines represented in each study were as follows: medicine (n = 18; 85.7%) [21,22,23,24,25,26,27,28,29,31,32,33,34,35,36,37,38,39], nursing (n = 15; 71.4%) [21,22,23,26,27,28,29,30,31,34,35,36,37,39,40], pharmacy (n = 14; 66.6%) [22,24,25,26,27,28,31,32,34,35,36,37,38,39] and social work (n = 13; 61.9%) [24,27,28,31,32,33,34,35,36,37,38,39,41]. Other professions included were physical therapy (n = 9; 42.8%) [24,25,26,28,32,34,39,40,41], occupational therapy (n = 7; 33.3%) [22,24,26,28,32,36,40], dietetics/nutrition (n = 5; 23.8%) [24,26,28,34,39], dentistry/dental hygiene (n = 3; 14.3%) [26,32,39], speech therapists (n = 2; 9.5%) [28,30], physician assistants (n = 2; 9.5%) [32,34], osteopathic medicine (n = 1; 4.8%) [30], psychology (n = 1; 4.8%) [37], public health (n = 1; 4.8%) [30], and others (n = 4; 19.0%) [21,27,28,30]. Overall, between two and ten different professions were included in CGA trainings within the studies of this review.

Applied educational and training interventions showed a high degree of heterogeneity, ranging from training courses [21]; a Geriatric Inter-professional Assessment Clinic for learners [24]; rotation [25] and simulation exercises [26,31]; implementation of inter-professional curricula [23,27] or inter-professional education models [28]; web-based block exercises [35]; inter-professional learning experiences [33,40]; inter-professional geriatric team exercises [37]; standardized patient exercises [39]; and general inter-professional education and training programs [22,29,30,32,34,36,38,41].

Primary outcomes of interest varied as well, with studies analysing overall inter-professional learning experience [22,24,37,39]; attitudes towards inter-professional practice and/or older adult care [28,36,38]; confidence in geriatric skills [25,27]; competencies [26,31]; inter-professional practice [33] or geriatric knowledge [29]. Other studies examined various combinations of data, such as attitudes, knowledge and practices [21]; confidence, skills and attitudes [34]; knowledge, competencies and patient health outcomes [41]; knowledge and competence [35]; knowledge and attitudes [32]; attitudes and skills [30]; attitudes and team performance [40] or specified primary endpoints within a randomized controlled trial [23].

The majority of the studies used a self-assessment approach of learners [21,25,26,27,28,30,31,32,33,34,36,37,38,40,41], with some publications applying more objective assessment measures [23,29,35] and the remaining ones using a combination of learners’ self-assessment with faculty [24,39] and residents’ evaluation [22].

### (3.2) Results from the quality assessment

The mean MERSQI score for the included studies was 10.52 (SD 2.07) with a score range from 6.5 to 15.0. Results for each domain indicate a mean score of 1.43 (SD 0.44) for study design, 1.83 (SD 0.51) for sampling, 1.57 (SD 0.85) for type of data, 1.45 (SD 1.13) for validity of evaluation instrument, 2.69 (SD 0.52) for data analysis and 1.50 (SD 0.51) for outcome domain.

### (3.3) Effectiveness of educational interventions

**Figure 2:**
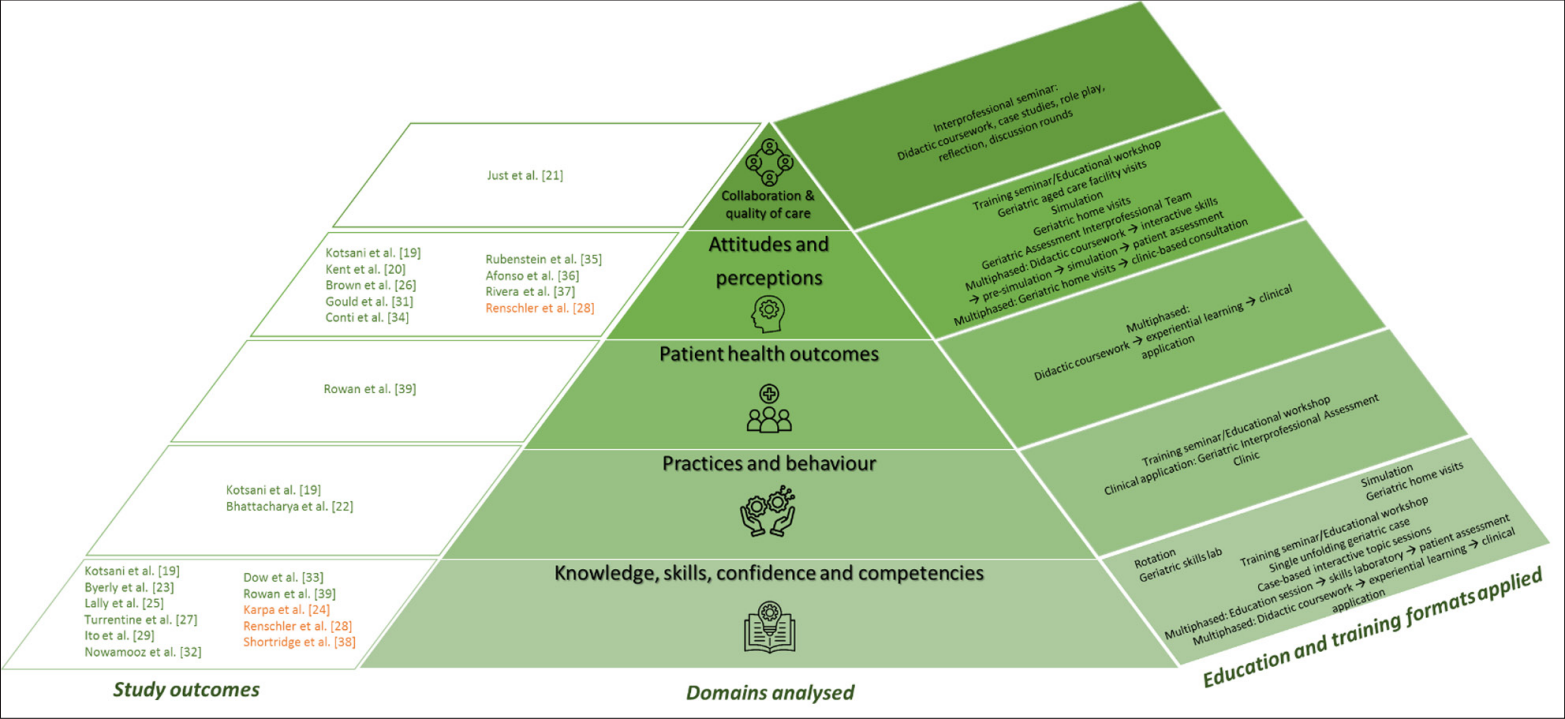
**Domains analysed, education and training formats applied and study outcomes.** Inspired by Miller‘s pyramid [42,43], Figure illustrates the domains analysed (central column), respective education and training formats applied (right column) and measures of effect for each study included (left column), indicating evidence for positive outcomes (green) or mixed outcomes (orange). Images: flaticon.com.

Primary outcomes investigated in studies included in this review ranged from evaluation of general attitudes and perceptions of the learning experience; knowledge, skills, skill confidence and competencies; practices and behaviour; patient health outcomes as well as other particular measures such as collaborative environment, communication style and quality of care objectives. Figure 2 provides an overview of the domains analysed, the educational and training methodologies applied and evidence of included studies.

#### (3.3.1) Attitudes and perceptions

Attitudes and perceptions in the context of providing geriatric care in an inter-professional environment were analysed in nine studies [21,22,28,30,33,36,37,38,39]. Eight studies reported positive outcomes [21,22,28,33,36,37,38,39], whereas one study described mixed outcomes [30].

Positive effects were shown on the development of attitudes towards frailty syndrome and its management in daily clinic towards inter-professional collaboration [33] and practice [28] in geriatric care in general. Kent et al. [22] report positive experiences of learners across all domains analysed, describing student perceptions of the educational experience, with an additional high mean rating of older residents with regards to communication (4.41 on a 5-point scale, SD = 0.62) and a low mean in the consultation domain (2.02 on a 5-point scale, SD = 0.81). Over 90% of the residents were very satisfied or satisfied with the educational consultation while over 72% found it useful or very useful. Rubenstein et al. [37] demonstrated high means of post-intervention experience scores on a 7-point scale in the domains mission, meaningful purpose and goals (6.53 ± 0.65); general relationships (6.61 ± 0.47); team leadership (6.36 ± 0.64); general role responsibilities and autonomy (6.22 ± 0.37); communication and information exchange (6.17 ± 0.61); community linkages and coordination of care (6.23 ± 0.72); decision-making and conflict management (5.63 ± 0.57) and patient involvement (6.98 ± 0.57). Other studies report overall high agreement of learners that the educational intervention improves inter-professional team functioning as well as comfort, communication with clinical management and knowledge of older adults [36,38,39]. One study also presents faculty attitudes, agreeing that the educational intervention enhances learners’ understanding of the patient care roles of different professionals (M = 5.81 on a 6-point scale, SD = 0.40) and that the intervention fosters communication between health professions students (M = 5.83, SD = 0.38) [39].

Mixed results are reported by Renschler et al. [30] who found no significant post-intervention improvement in attitudes of osteopathic medical students (p = 0.07–0.99). However, attitudes improved significantly in subscales for nursing, public health and allied health students (p = 0.00–p = 0.01).

#### (3.3.2) Knowledge, skills, confidence and competencies

Eleven studies investigated the domains knowledge, skills, (skills) confidence and competencies [21,25,26,27,29,30,31,34,35,40,41] with eight studies demonstrating positive outcomes [21,25,27,29,31,34,35,41] and three studies reporting mixed outcomes [26,30,40].

Turrentine et al. [29] describe a significant post-intervention improvement of geriatric knowledge (p < 0.001) and a frailty training course conducted in Greece resulted in significant post-intervention improvement of knowledge and skills to recognize and manage frailty (p < 0.001). This improvement was still observed three months after the intervention (p = 0.001 for frailty recognition and p = 0.003 for frailty management) [21]. Dow et al. [35] analysed geriatric knowledge, student and team competencies following a six weeks block with a single unfolding case in geriatric care. Post-intervention knowledge scores were the highest for medical students (M = 3,918; SD = 996), followed by nursing students (M = 3,462; SD = 825) and pharmacy students (M = 3,119; SD = 1,431) whereas social work students scored the lowest (M = 1,454; SD = 901). Knowledge team scores were significantly higher than individual scores in all professions (p < 0.001). In a Geriatric Evaluation and Self-Management Service Training conducted in the USA [41], mean post-intervention knowledge scores were 82.22% (SD = 7.96) for the domain self-management, 86.94% (SD = 10.48) for assessment and 90.20% (SD = 7.34) for telehealth. Learners exhibited moderate to high confidence to assess, intervene and function within an inter-professional team, whereas participating professionals indicated a significant higher rated confidence to assess than students did. Competency scores revealed no significant difference among disciplines. Other studies reported a significant post-intervention increase of skills [31] or (skills) confidence [25,27,34]. In detail, learners evaluated by Ito et al. [31] demonstrated a significant post-intervention increase of all skills assessed, containing values and ethics (p = 0.0004–p = 0.002); roles and responsibilities (p < 0.0001); communication (p < 0.0001) and teamwork (p < 0.0001). A significant post-intervention improvement in confidence in 11 of 12 items (p < 0.001–p < 0.05) was determined by Lally et al. [27], this corresponds with results found by Byerly et al. [25], reporting a significant post-intervention improvement of confidence in geriatric skills (p < 0.001). Moreover, the rotation intervention increased learners’ skill set in geriatric care (M = 4.7 on a 5-point scale, SD = 0.46) and inter-professional aspects of care planning positively contributed to the overall educational experience (M = 4.7, SD = 0.53). An inter-professional geriatric education program led to an increased confidence in conducting MMSE (95%); falls risk assessments (93%); medication reviews (78%); and understanding of team collaboration in favour of patient care and safety (81%) [34].

Mixed results are reported in terms of (skills) confidence [40], self-assessed competencies [26] and teamwork skills [30]. A multi-phased learning experience based on a standardized patient encounter resulted in 50% of students expressing confidence in care provision of their respective teams, however, 29% of the participants raised concerns in free comments. Level of confidence differed across disciplines with nurse practitioner students feeling most confident while physical therapy students expressing lowest confidence levels [40]. Although Karpa et al. [26] reported a cumulative significant post-intervention improvement in self-assessed interaction competencies (p = 0.04), no significant post-intervention improvement was found in individual items. Renschler et al. [30] found a significant post-intervention improvement in teamwork skills for nursing, public health and allied health students in the short training program (p = 0.00) and long training program (p = 0.01), however, no significant improvement of teamwork skills was found for osteopathic medical students, also both in the short (p = 0.14) and long training (p = 0.07) program.

#### (3.3.3) Practices and behaviour

Everyday practices and team behaviour were analysed by two studies included in this review [21,24]. The educational workshop as reported by Kotsani et al. [21] resulted in substantial adaptations in everyday practices with a significant post-intervention frequency of frailty screening tools (p = 0.014) and 70% of participants declaring the intention to modify their daily practice. Three months after the educational intervention, 32% modified their practice and 36% moderately modified their practice. 40% of the participants applied frailty management strategies three months after the intervention, interventions offered post training being positively perceived by 60% of older patients cared for and 90% of their families. An educational experience within a Geriatric Inter-professional Assessment Clinic resulted in significant improvements to patient education and communication of the care plan (p = 0.023) and consideration of patients’ expectations (p = 0.013). All team behaviour items demonstrated a positive change from early evaluations at intervention start to late evaluations towards the end of the rotation [24].

#### (3.3.4) Patient health outcomes

Patient health outcomes were analysed by one study and are briefly presented here [41]. Considered health parameters were self-efficacy, self-rated health, functional status, physical mobility and mental health. After providing comprehensive, inter-professional assessments and self-management care plan recommendations by the health care teams, older adults showed significant health-related benefits and all outcome parameters demonstrated a positive change [41].

#### (3.3.5) Other outcomes

In terms of collaboration and quality of care, a randomized controlled trial conducted with medical and nursing students reported a post-intervention increase of evaluated care objective scores in the intervention and control group in all but one category: care of other symptoms. The intervention group achieved significant post-intervention scores in three categories: pain therapy (p = 0.006); guarding of patient’s autonomy (p = 0.001) and integration of psychological aspects (p = 0.003). Moreover, a significant change in interaction initiation was observed in the intervention group (p = 0.0007) and there was a significant post-intervention increase in the number of uni-professional information items exchanged in both groups (p < 0.0001) and in subgroups (p = 0.0002-p = 0.004) [23].

Byerly et al. [25] additionally evaluated the extent of which inter-professional learners perceived their environment as supportive for collaboration. Team average scores ranged from 46.0 (SD = 2.98) to 59.7 (SD = 0.45) on a 60-point scale, indicating that the teams regarded the educational environment as collaborative.

## (4) Discussion

Definition and development of transversal skills of tomorrow’s health workforce will be key to enable different professions in health and social care to deliver high quality and patient-centred integrated care in health care systems of the future [1,44]. Skills for handling complex tasks and creating a positive working culture will be essential for future staff to be able to provide personalized care to ageing societies. Working aside in a technically sophisticated healthcare environment asks for smooth management of complex personal relations with an inter-professional team, equally important to the need of team resilience and support. Very recently, van Wijngaarden and colleagues presented a scoping review for delivery of under- and postgraduate training programs for medical doctors (MDs) to prepare them for delivery of this “integrated care for elderly” [45]. They demonstrate evidence, that exposure of students and residents to complex, reality-based care situations increased students’ understanding of integrated care processes. Moreover, exposure to complex task solving during interprofessional trainings has been demonstrated to improve clinicians’ capacity to develop “adaptive expertise” during their whole professional career, allowing straightforward application of their knowledge and transversal skills, such as collaboration, communication and positive team dynamics, particularly in situations of novelty and complexity [46]. In this context of integrated care for older people, CGA may be seen as “the core concept” and requires a high level of adaptive expertise from different professions involved into the person-centred care for older patients.

Based on the concept of ICP and “adaptive expertise”, the current paper describes the evidence-base for trainings offered for different health professional groups to perform CGA in inter-professional collaborative practice.

The overall care rendered by CGA teams providing longitudinal assessment and care can be divided into six steps: data-gathering, discussion among the team, increasingly including the patient and/or caregiver as a member of the team, development of a treatment plan – together with the patient and/or caregiver, implementation of the treatment plan, monitoring response to the treatment plan and finally revising the treatment plan [47]. Given the framework of this inter-professional process, CGA has been shown to improve outcomes for older people in hospital and community settings [7] as it encompasses health and social care needs and facilitates multidisciplinary working. However, supporting health and social workers to achieve the collaborative capacity to deliver personalized care raises the need to acquire skills and attitudes enabling staff to work jointly in daily practice. Besides professional standards and ethics, a strong attitude towards patient relations, inter-professional team working, redefinition of roles and relations and/or communication build the ground for an integrated management and organization flow when offering CGA to older people and patients. Understanding how to best equip todays health and social care staff with these core competencies remains a challenge up to this point [48]. Some of the studies included indicated a variability in results according to profession [32,36]. This may underline the necessity of IPE to manage the shift from an existing professional identity towards identification and definition of one’s role within an interprofessional team [49].

The need for targeted inter-professional education and training initiatives in geriatric care has been highlighted by literature for some time [50,51,52]. Indeed, the concept of IPE offers the potential to deliver optimized patient-centred health outcomes and contributes to collaborative patient care [53,54]. Education of inter-professional skills and competencies cannot be transmitted via lectures or traditional classroom-teaching but rather needs to be learned in a practice-oriented setting [40], as inter-professional learning is composed of experiential learning as well as social learning, indicating that the experiences and social activities students encounter during their educational interventions are direct “products” of the learning process itself [53].

Studies included were of varying quality in study design, only one study using a randomized controlled trial approach design [23]. Duration of training interventions ranged from 1-day courses [21] to training modules of 19 months [41]. Despite a clear process description within the internationally agreed format of CGA, none of the publications took into consideration a step-to-step evaluation of the six steps foreseen for CGA, from collective data gathering to monitoring and revision of interventions. All studies included focused on transversal skills gain as inter-professional training results thereby reflecting only a short window of outcome evaluation opportunity when aligned with educational evaluation standards, such as the model of Kirkpatrick [55]. This model of learning evaluation describes four different levels, starting from learners’ reaction to education and training initiatives (Level 1), actual learning in terms of knowledge, skills or experience increase (Level 2), impact on learners’ behaviour in daily work (Level 3) and results on organizational level (Level 4) [55].

Given the heterogeneity of IPE interventions published in the papers included and this major weakness in the evaluation frameworks applied to evaluate the educational impact of training interventions, the data provided in this review should be reflected thoroughly. Two of the studies included [28,39] set up a training framework mimicking the six step approach underlining the process of CGA. Unfortunately, both studies did not follow the step-to-step approach in their evaluation approach. Brown et al. [28] tested self-perceived change in students’ attitudes in different domains, whereas Rivera and colleagues [39] used a single arm post-test evaluation design in self-assessment and faculty feedbacks. This approach covers only part of the learning outcomes targeted by the educational intervention described in the papers and therefore does not allow a solid evaluation and/or recommendation of the impact of educational offers.

Results of this review need to be considered in light of its limitations. As already mentioned, the studies included present with a strong heterogeneity in terms of study design, outcomes and evaluation methodologies. This hampers the possibility to present a clear evidence-base and only allows drawing a cautiously positive picture of effectiveness of educational interventions to train CGA across health and social care professions, which is aggravated by the fact that not all studies provided full disclosure of educational content delivered and intervention execution. Most of the studies included presented self-reported data based on learners’ perceptions instead of objective assessment results or patient health outcomes that may lead to questioning reliability and generalizability of effects.

Despite the mentioned heterogeneity, the majority of studies included encompasses didactic approaches with a strong practical orientation, either in form of real patient encounters [22,24,25,30,32,33,36,37,38,41] or standardized patient simulations [26,28,29,31,39,40]. Other studies applied (unfolding) case vignettes [23,35]; role-play sessions [27] or workshops with limited practical reference [21]. These results may be seen in alignment with previously published data on trainings for integrated care [45].

The data on training formats, however, contrast with assessment methods applied as only two studies assessed practices and behaviour [21,24] and only one study additionally assessed patient related health outcomes [41].

None of the studies used the Objective Structured Clinical Examination (OSCE) as assessment methodology. The OSCE is a widespread method and gold standard to assess clinical skills and competencies transmitted during educational and training initiatives as objectively as possible [56,57]. Despite the fact that this assessment approach enables a standardized way to examine clinical competencies of students and learners, it also does not necessarily ensure reliability and objective judgment [58]. Moreover, there is discussion regarding research on students’ self-assessment arguing that self-assessment methodologies offer the great possibility for students to deepen their self-engagement in the learning progress. If formal and standardized education programs should address needs and goals for future healthcare workforce education [13,59] properly, there needs to be a clear understanding and consensus among experts and educators on roles and competences of different professions involved. Frameworks defining required core competencies of an inter-professional health and social care workforce may aid in targeted curriculum development in this regard and contribute to facilitation in delivery of inter-professional collaborative practice for an ageing population [15,60,61].

Although authors of this review adhered to guidelines for writing systematic reviews [18,19], some limitations concerning research techniques need to be acknowledged. Despite a comprehensive search strategy including grey literature on Google scholar and reference tracking, some relevant studies may have been missed. This is possibly due to language barriers as only studies published in English and German were included, while IPE has emerged as a global movement, often responding to locally emerging needs [62]. Considering that none of the studies included presented results with a neutral or negative outcome, authors need to be aware of the well-recognized publication bias, keeping in mind that research endeavours with negative outcomes may not be published to a comparable extent. Finally, authors would also like to point out that critical appraisal of the studies included is mostly based on subjective reasoning. Even though the applied MERSQI tool [20] represents an easy-to-use, standardized instrument, ratings are primarily based on individual judgments and interpretation.

## (5) Conclusion

This systematic review highlights effects and possibilities to educate and train professionals in health and social care in conducting a CGA and shows promising results for inter-professional collaborative practice in geriatric care, when measured with students’ self-assessment scales. There seems to be potential for introducing case-based and/or work-placed teaching methodologies reflecting the complexity of interacting physical, psychological/emotional, contextual and social factors evaluated during CGA for different professions, enabling a shared decision process during CGA of older people.

However, further educational research, preferably reflecting effects, needs and progress of interprofessional collaborative practice in the European education/training landscape and focussing on effectiveness of complex care based training situations based upon standardized educational assessment frameworks is needed to strengthen the evidence-base, foster education and training standards as well as curricular development in an inter-professional context and thus adequately equip the health workforce to acquire adaptive expertise for delivery of CGA and, therefore, meet the needs of an ageing population.

## Additional File

The additional file for this article can be found as follows:

10.5334/ijic.7549.s1 Supplemental material. Research protocol.
