# Supplementary material for: Effectiveness of multi-professional educational interventions to train Comprehensive Geriatric Assessment (CGA) – a Systematic Review
Source: Int J Integr Care. 2023 Aug 14;23(3):9. doi: 10.5334/ijic.7549 (PMC10437136; doi:10.5334/ijic.7549)
Supplement: Supplemental material. — Research protocol. [file ijic-23-3-7549-s1.pdf]

Study Characteristics

Author/Year	Country	Study Design	Study Population (and subgroups) description	Education/Training Intervention	Primary outcomes of interest and evaluation tool	Primary measures of effect
**Kotsani 2021**	Greece	Quasi experimental study (single arm pre-/post-test)	Family medicine general practitioners, general internal medicine specialists, nurses, health visitors<br>Pre-evaluation: n=31<br>Post-evaluation t1 (after intervention): n=30<br>Post-evaluation t2 (3 months later): n=25	• 1-day training course on detection, first-line assessment and management of frailty	• *Self-assessed attitudes, knowledge and everyday practices* (no further information available)	• Significant post-intervention improvement of self-assessed knowledge and skills to recognize and manage frailty immediately (p<0.001) and also 3 months later (p=0.001 for recognition; p=0.003 for management)<br>• Significant post-intervention frequency of use of screening tools for frailty (p=0.014)<br>• 76.7% of participants reported a significant or large and the rest a moderate impact of the intervention on their attitudes toward the frailty syndrome<br>• 76.7% estimated a high or very high usefulness in clinical practice after the workshop<br>• 70% reported that they would be quite or very likely to proceed to modifications in their daily practice<br>○ 3 months later, 32% did modify their practice, 36% did so only moderately<br>• 40% applied frailty management strategies 3 months after the intervention, that were positively perceived (health care services satisfaction) by 60% of older patients and 90% of older patients' families
**Bhattacharya 2021**	USA	Quasi experimental study (single arm pre-/post-test)	Medicine, occupational therapy, pharmacy, dietetics, physical therapy and social work students (n=247)	• Geriatric Interprofessional Assessment Clinic	• *Interprofessional team behaviour* (Development of the Standardized Patient Encounter Evaluation Rubric – SPEER Tool)	• Learners scored themselves significantly higher than faculty scores (p<0.001)<br>• All items had a positive relative change from "early" to "late" team evaluations<br>• Significant improvements from pooled "early" to "late" faculty evaluations for the team's ability to allow all members of the team to contribute to communicating the plan to the patient (p=0.023)<br>• Significant improvements in learner evaluations for the team allowing the patient to voice their expectations of the team visit at the beginning of the visit (p=0.013)

Study Characteristics

**Byerly 2020**	USA	Quasi experimental study (single arm pre-/post-test)	Senior medical, pharmacy and physical therapy students (n=31)	• 10-day interprofessional nursing facility rotation	• *Self-reported confidence in geriatric skills* (no further information available)<br>• *Collaborative Environment* (Based on Tilden et al.: Development of the Assessment for Collaborative Environments (ACE-15): a tool to measure perceptions of interprofessional “teamness”)	• Significant post-intervention improvement in confidence in geriatric skills (p<0.001)<br>• Intervention increased skill set in geriatric care (M=4.7 out of 5, SD=0.46) and interprofessional aspects of care planning activities added to participants’ overall educational experience (M=4.7, SD=0.53)<br>• Team overall average scores of the collaborative environment (Average scores ranged between 46.0/SD=2.98 and 59.7/SD=0.45 out of 60 per team) suggested that the skilled nursing facilities environment was conducive for interprofessional teamwork
**Karpa 2019**	USA	Quasi experimental study (single arm pre-/post-test)	Second-year medical students, first-year nursing students, second-year occupational therapy students, second-year physical therapy students, third-year pharmacy students, second-year dental hygiene students, dietetic students (n=10)	• 3-hour interprofessional simulation about comprehensive geriatric assessments using standardized patients	• *Self-assessed competencies* (Questionnaire, based on Lockeman et al.: Refinement of the IPEC Competency Self-Assessment survey: results from a multi-institutional study)	• Significant post-intervention improvement in self-assessed interactions competencies (cumulative) (p=0.04)<br>• No significant post-intervention improvement in individual items
**Lally 2018**	USA	Quasi experimental study (single arm pre-/post-test)	Nurses, nurse practitioners, social workers, pharmacists, physicians, others (trainees)<br><br>Goals of care – Table top training Evaluation: n=40<br><br>Goals of care – Role play training Evaluation: n=38<br><br>Geriatric Assessment training Evaluation: n=28	• Curriculum development in palliative care and geriatrics	• *Skills confidence* (Adapted from Chow et al.: We can do it: Nursing educational intervention to increase delirium documentation; and Leopold et al.: Impact of educational intervention on confidence and competence in the performance of a simple surgical task)	• Significant post-intervention improvement in confidence in 11 of 12 items (p<0.001-p<0.05)
**Brown 2018**	USA	Quasi experimental study (single arm pre-	Pharmacy, medicine, nursing and allied health (speech, physical, occupational therapies, nutrition, social	• Interprofessional Education Model	• *Attitudes associated with interprofessional practice* (Adapted from King et al.: The interprofessional	• Significant post-intervention improvement of attitudes in all subscales and total scores (p<0.001)

		/post-test)	work, counselling, chaplain, Emergency Medical Tech) students (n=136)		socialization and valuing scale: a tool for evaluating the shift toward collaborative care approaches in health care settings)	
**Nowamooz 2018**	USA	Quasi experimental study (single arm post-test)	Medicine, physician assistant, physical therapy, pharmacy, nursing, social work and dietetics students (n=718)	• Interprofessional geriatric education program: Seniors Assisting in Geriatric Education	• *Self-assessed confidence, skills and attitudes* (no further information available)	• Post-intervention increased confidence in conducting MMSE (95%), falls risk assessments (93%), medication reviews (78%), and understanding of team collaboration to improve patient care and safety (81%)
**Turrentine 2016**	USA	Quasi experimental study (single arm pre-/post-test)	Fourth-year medical and nursing students (n=15)	• Interprofessional training	• *Geriatric knowledge* (no further information available)	• Significant post-intervention improvement of geriatric knowledge ($p<0.001$)
**Dow 2016**	USA	Quasi experimental study (single arm post-test)	Fourth-year medical, pharmacy and nursing students; second-year social work students (n=522)	• Six-weeks block web-based case system	• *Geriatric knowledge* (Based on Moore et al.: Achieving desired results and improved outcomes: Integrating planning and assessment throughout learning activities)<br>• *Student and team competence in interprofessional practice and Electronic Health Record use* (Based on Moore et al.: Achieving desired results and improved outcomes: Integrating planning and assessment throughout learning activities)	• Post-intervention knowledge scores were highest for medical students (M=3,918; SD=996), followed by nursing students (M=3,462; SD=825) and pharmacy students (M=3,119; SD=1,431) whereas social work students scored the lowest (M=1,454; SD=901).<br>• Post-intervention knowledge team scores were significantly higher than individual scores for all professions ($p<0.001$)
**Conti 2016**	USA	Quasi experimental study (single arm post-test)	Medical, bachelor-level nursing, second- and third-year pharmacy and occupational therapy, bachelor- and master-level	• Interprofessional education activity	• *Attitudes towards older adults, interprofessional education team functioning and value of home visits* (Based on	• Overall mean agreement to all likert-based survey statements was 88%<br>• Students agreed on following statements:<br>○ The intervention improved the opinions about other healthcare

Study Characteristics

			social work students (n=861)		Learning from Interprofessional Educational Experiences – LIPEE survey)	professionals (92.6%)<br>○ The intervention experience would make them more effective to work as a member of a healthcare team (93.8%)<br>○ The intervention improved communication with other healthcare professionals (92.5%)<br>○ The intervention improved comfort of interaction with older adults (86.0%)<br>○ The intervention helped to understand clinical problems (87.4%) and older adults' problems (85.8%)<br>○ The intervention helped to communicate better with older adults (80.5%)<br>○ The intervention increased understanding of the importance of respect, dignity and privacy (92.8%)<br>○ Education in team-working skills is vital (98.9%)
**Renschler 2016**	USA	Quasi experimental study (multi arm pre-/post-test)	Athletic training, speech-language pathology, exercise sciences, public health, nursing, and osteopathic medicine students (n=148)	• 1-semester or 2-semester Interprofessional Geriatric Home Visit Program	• *Attitudes towards interprofessional healthcare teamwork skills* (Based on Attitudes Towards Healthcare Teams Scale – ATHTC)<br>• *Teamwork Skills* (Based on Team Skills Scale – TSS)	• No significant post-intervention improvement in attitudes and teamwork skills for the 1-semester and 2-semester programme for osteopathic medical student participants (p=0.07 – p=0.99)<br>• Significant post-intervention improvement of attitudes in 3 out of 4 subscales in the 1-semester programme for nursing, public health, and allied health student participants (p=0.00 – p=0.01)<br>• Significant post-intervention improvement of attitudes in one subscale in the 2-semester programme for nursing, public health and allied health student participants (p=0.00)<br>• Significant post-intervention improvement in teamwork skills for the 1-semester (p= 0.00) and 2-semester programme (p=0.01) for nursing, public health and allied health student

Study Characteristics

						participants
**Kent 2016**	Australia	Quasi experimental study (single arm post-test)	Fourth and final year medical, nursing, occupational therapy and pharmacy students (n=26)	• 1-2 afternoon Interprofessional student teams in an residential aged care facility	• *Students' perception of the learning experience* (Adapted from Roff S.: The Dundee Ready Educational Environment Measure (DREEM) – a generic instrument for measuring students' perceptions of undergraduate health professions curricula<br>• *Residents' experience* (Based on Steine et al.: A new, brief questionnaire (PEQ) developed in primary health care for measuring patients' experience of interaction, emotion and consultation outcome)<br>• *6-weeks follow-up of residents for reflective view of consultation by interview*	• Post-intervention mean ratings of students' perception indicate positive experiences across all domains (mean range: 4.04; SD=0.66 – 4.62; SD=0.57)<br>• Residents' perceptions achieved a low mean on a 5-point scale of 2.02; SD=0.81 in the consultation outcome domain and the highest mean of 4.41; SD=0.62 in the communication domain<br>• 6-weeks follow-up resulted in 10/11 residents being satisfied or very satisfied with the consultation, while 8/11 found it useful or very useful
**Shortridge 2016**	USA	Quasi experimental study (single arm post-test only and pre-/post-test)	Nurse practitioner, physical therapy and occupational therapy students<br>Team-performance post evaluation: n=86<br>Attitudes toward telehealth pre-post evaluation: n=85	• Multiphased learning experience	• *Reflection on team-performance* (Based on Zimmerman, B.: Investigating self-regulation and motivation: historical background, methodological developments, and future prospects)	• 50% of students' statements expressed confidence in care provision of their teams, 29% raised concerns<br>• Differences in level of confidence according to disciplines: Nurse practitioner students felt most confident, physical therapy students the least confident
**Rubenstein 2016**	USA	Quasi experimental study (single arm post-test)	Nurse, social worker, pharmacy resident, doctoral psychology student, family medicine resident, geriatrics fellow, geriatrics faculty	• Geriatric Assessment Interprofessional Team (GAIT)	• *Intervention Experience* (Based on Collaborative Practice Assessment Tool – CPAT)	• Post-intervention average score for each domain (maximum score 7): Mission, Meaningful Purpose, Goals 6.53±0.65; General Relationships 6.61±0.47; Team Leadership 6.36±0.64; General Role Responsibilities,

Study Characteristics

			(n=10)			Autonomy 6.22±0.37; Communication and Information Exchange 6.17±0.61; Community Linkages and Coordination of Care 6.23±0.72; Decision-making and Conflict Management 5.63±0.57; and Patient Involvement 6.98±0.57
**Ito 2016**	USA	Quasi experimental study (single arm retrospective pre-/post-test)	Third-year medical, nursing, pharmacy and social work students (n=104)	• Hawaii Interprofessional Team Collaboration Simulation	• *Self-rated interprofessional collaborative practice core competencies* (no further information available)	• Significant post-intervention increase for all eight self-assessed skills, including competencies of Values/Ethics (Q1 4.44 vs. 4.69, p=0.0004; Q2 4.45 vs. 4.62, p=0.002), Roles/Responsibilities (Q3 3.78 vs. 4.33, p<0.0001; Q4 3.85 vs. 4.42, p<0.0001), Communication (Q5 3.89 vs. 4.37, p<0.0001; Q6 3.90 vs. 4.41 p<0.0001), and Teamwork (Q7 3.96 vs. 4.40, p<0.0001; Q8 4.03 vs. 4.49, p<0.0001)
**Reilly 2014**	USA	Quasi experimental study (single arm pre-/post-test)	Second-year medical, physician assistant, occupational therapy, social work and physical therapy students; third-year pharmacy students; fourth-year dental students (n=67)	• Interprofessional, team-based, geriatric home training program	• *Knowledge and attitudes towards interprofessional education* (Adapted from Readiness for Interprofessional Learning Survey)	• Significant post-intervention change in one of 19 items for all students: improved understanding of role in an interprofessional health care team (p<0.001)<br>• Significant post-intervention changes in some particular items within each discipline
**Gould 2014**	USA	Quasi experimental study (single arm pre-/post-test)	Medical and social work graduate students (n=18)	• Interprofessional learning experience	• *Perception of interprofessional collaboration* (Based on Bronstein, LR.: Index of Interdisciplinary Collaboration)	• Increased post-intervention level of interprofessional collaboration<br>• Significant post-intervention changes to participants' perception of interprofessional collaboration (Z= - 2.202, p=0.028)<br>• Significant post-intervention changes in subscale "reflection on process" (Z= - 2.190, p=0.029)
**Afonso 2013**	USA	Quasi experimental study (single arm post-test)	Medical, second- and third-year pharmacy, bachelor- and master-level social work students (n=941)	• Experiential Interprofessional Education using an older adult home visit	• *Attitudes towards Interprofessional Education* (no further information available)	• Students agreed on following attitudes:<br>○ Interprofessional teams are more likely to delivery comprehensive care (97.1%)<br>○ Interprofessional teams would improve health outcomes for older adults (99.0%)

Study Characteristics

						○ Ability to work in a multidisciplinary team contributes to professional success (90.7%)<br>○ Intervention gave them an increased understanding of the role of other team members (92.0%)<br>○ Home visits generate more patient information (90.4%) and increased knowledge of older adults (86.2%)<br>○ Intervention made them feel more comfortable with older adults (75.3%)
**Rivera 2013**	USA	Quasi experimental study (single arm post-test)	Third- and fourth-year dentistry, medical, pharmacy and physical therapy students; Second-year nurse practitioner and social work students; dietician interns (n=261)	• 3-hours Interprofessional Standardized Patient Exercise	• *Intervention Experience of students and faculty* (no further information available)	• Students agreed on following statements (6-point scale):<br>○ They learned about the patient care roles of other health care professionals (M=5.22; SD=0.96)<br>○ They increased their comfort working collaboratively in an interprofessional team (M=4.99; SD=1.04)<br>○ Would recommend the intervention to fellow trainees (M=5.09; SD=1.05)<br>• Faculty agreed on following statements (6-point scale):<br>○ Intervention enhances student understanding of the patient-care roles of different professionals (M=5.81; SD=0.40)<br>○ Intervention fosters communication between participating health professions students (M=5.83; SD=0.38)<br>○ Would recommend the intervention to learners (M=5.96; SD=0.20)
**Just 2010**	Germany	Randomized Controlled Trial (RCT)	Third-year medical and nursing students (n=40)	• Intervention group: 2-day Interprofessional Curriculum<br>• Control group: Written material	• *Uni-professional items exchanged during interaction*<br>• *Communication style*<br>• *Quality of care objectives*	• Significant post-intervention increase in the number of information items exchanged for both the control group and the intervention group in total (p<0.0001) and for the medical (control: p=0.0004; intervention: p=0.004) and nursing subgroups (p=0.0002)

				with content of curriculum		• Significant post-intervention change in interaction initiation in the intervention group (p=0.0007)<br>• Care objectives score increased post-intervention for both groups in all but one category: care of other symptoms<br>• Significant post-intervention increase in score of control group in two categories: guarding of patient's autonomy (p=0.001) and integration of psychological aspects (p=0.001)<br>• Significant post-intervention increase in score of intervention group in three categories: pain therapy (p=0.006), guarding of patient's autonomy (p=0.001) and integration of psychological aspects (p=0.003)
**Rowan 2009**	USA	Quasi experimental study (single arm post-test and pre-/post-test)	Master-level social work students and professionals, and physical therapy students and professionals (n=66)	• 19-month Geriatric Evaluation and Self-Management Services Training	• *Knowledge* (Post-test only; Created for study, no further information available)<br>• *Competencies* (Post-test only; Geriatric Social Work Competency Scale II)<br>• *Client Outcomes* (Pre- and Post-test; Self-efficacy, self-rated health, functional status, physical mobility, mental health)	• Mean-percentage score was 82.22% (SD=7.96) for self-management post-test, 86.94% (SD=10.48) for assessment post-test and 90.20% (SD=7.34) for telehealth post-test<br>• Moderate to high confidence to assess, intervene and function as an interdisciplinary team<br>• Significant higher rated confidence to assess of professionals than of students<br>• No difference in confidence to intervene or function as an interdisciplinary team between professionals and students<br>• No significant difference between disciplines on competency scores<br>• Significant health-related benefits for older adults
